# Using patient perspectives to inform communication training materials for health care professionals discussing BRCA mutation testing

**DOI:** 10.1007/s10549-020-05871-4

**Published:** 2020-08-18

**Authors:** Valerie Shilling, Susan Catt, Valerie Jenkins, Lesley Fallowfield

**Affiliations:** Sussex Health Outcomes Research and Education in Cancer (SHORE-C), Brighton and Sussex Medical School, University of Sussex, Falmer, Brighton, BN1 9RX UK

**Keywords:** Communication, Genetic testing, BRCA, Breast cancer, Qualitative

## Abstract

**Purpose:**

As demand for genetic testing grows and a wide range of health care professionals (HCPs) are potentially involved in discussions about testing and delivering results, we developed an educational package to help HCPs with these conversations.

**Methods:**

To inform the content of training materials, we conducted interviews with 11 women four of whom had BRCA1 and seven with BRCA2 mutations. Five women had or were currently receiving breast cancer treatment. Ages ranged from 38 to 77 years. Interviews were audio-recorded, transcribed verbatim and analysed using the Framework approach to thematic analysis.

**Results:**

We identified 18 themes and 12 subthemes across the interviews, encompassed by six overarching themes: risk, decision-making, information and understanding, communication and improvement, accessing the system: process and frustration, emotional and social drivers.

**Conclusions:**

The findings informed the didactic components of an educational communication workshop and a summary document for attendees. Qualitative interviews provide an important way of incorporating the patient perspective into communication training materials for HCPs by highlighting key issues that matter most to the patient.

## Introduction

BRCA1 or 2 mutations may convey lifetime (to age 80) risks of breast cancer of up to 72% and 69%, respectively, and 44% and 17% risks of ovarian cancer [1]. Demand for accessible testing is growing but genetic services are operating under rapidly increasing strain and demand may exceed the availability of counselling services. As a result, many different healthcare professionals (HCPs) are potentially involved in discussions about the need for genetic testing, the consequences of a test result and implications for other family members [2, 3].

To provide genetic risk assessment, testing, and counselling, HCPs need a sound knowledge base, good communication skills and an ability to offer appropriate psychosocial support [4–6]. They must be able to interpret and then clearly convey risk information together with the implications this has for referral and management options [7]. This can be particularly challenging as many HCPs struggle when explaining numerical data [8]. Checking understanding and tailoring the discussion accordingly is essential [6, 7, 9, 10] while ensuring adequate detail is provided to meet standards for informed consent [11]. Recognition of the emotional context is also crucial, but such concerns are not consistently explored [12].

Given the difficulties inherent in communication about genetic risk, it is unsurprising that HCPs without specific genetic training may lack confidence when having such discussions [13, 14]. Some communications guidance is available for clinicians [15], but most HCPs would benefit from more focussed and specific training in this area.

### The TRUSTING educational programme

We developed the TRUSTING (Talking about Risk, Uncertainties of Testing IN Genetics) educational programme in close collaboration with experienced doctors, genetic counsellors, geneticists and patients, to expand the communication skills of all HCPs now involved in genetic risk discussions.

TRUSTING workshops contain a mix of didactic presentations, exercises and facilitated group discussions around filmed scenarios created specifically for the programme; these show HCPs discussing risk and genetic testing in the context of breast cancer germline mutations with different members of a fictitious affected family. Topics covered during the workshops include research-based evidence about ways to present complex information and risk information, ethics, communication about family history, what genetic testing means for family members, discussing BRCA test results and implications with gene mutation carriers and those who test negative, together with the impact that health literacy and numeracy exerts on decision-making.

We describe here a qualitative interview study with women with or without a diagnosis of breast cancer, who tested positive for a BRCA1/2 mutation. Our primary objective was to explore their views and understanding about the genetic testing process and experiences when talking to HCPs. Results helped highlight key issues around communication, information needs and decision-making which were then used to develop some of the training materials for the TRUSTING programme.

## Materials and methods

### Participants

Women with a known BRCA 1/2 gene mutation who were over 18 years of age and able to speak and read English were eligible for the study. Women in the process of genetic testing without a known result were excluded.

Participants were recruited via BRCA support groups and family history clinics. The invitation was circulated by group coordinators; those interested contacted SHORE-C researchers directly. Potential interviewees were sent the Participant Information Sheet in advance of a telephone call from a researcher. Signed informed consent was received from all participants.

Though largely a sample of convenience, we purposely sampled women who already had a breast cancer diagnosis, together with others without breast cancer who had tested positive for a gene mutation and who may have had risk-reducing surgery.

### Interview topic guide and procedure

We conducted thematically focussed interviews around five broad topics identified a priori: discussion of risk and presentation of risk information; information needs and information provided; communication style and approach; understanding; decision-making. These key areas for discussion were identified through literature review, group discussion with local peer support groups for women with a positive BRCA1/2 diagnosis, and through discussion within the research team. Participants were, however, encouraged to speak freely, raising topics of importance to them even if these were not prespecified in the topic guide.

Interviews were conducted by an experienced qualitative researcher (one of the authors, VS or SC), face-to-face or by telephone, at a time and place convenient to participants depending on their preference and location. Duration was participant guided and lasted between twenty-five minutes to over an hour. All were audio-recorded and transcribed verbatim.

### Data analysis

We applied the Framework Approach (Ritchie and Spencer [16]) to thematic analysis, which is a systematic and rigorous methodology for applied qualitative research permitting the data to be interrogated with a priori areas of interest in mind, rather than being an entirely inductive approach [17].

The Framework Approach involves five distinct stages: (i) familiarisation with the data (ii) identifying a thematic framework—identifying the key concepts and issues both a priori and those emerging from the data of individual respondents and recurring concepts; (iii) indexing—applying the framework to the transcripts; (iv) charting—extracting data from its original context, summarising and grouping it in chart form according to the thematic reference; (v) mapping and interpretation.

Following the five stages of the Framework approach, two of the authors (VS and SC) familiarised themselves with the data, read the transcripts and established the thematic framework. The coding structure was developed incorporating both deductive elements, based on a priori areas of interest identified in the literature and the topic guide, and inductive components, as new themes emerged. Sentences, phrases or single words were used to generate codes for the identification of themes. VS indexed and charted the material. SC also indexed and charted 25% of the transcripts, allowing for checks to be made for comprehensiveness of data extraction, and consistency in the application of the index. Differences in interpretation were resolved through discussion. In effect, this process ensures that the thematic framework is an adequate and appropriate way to group the data and that themes and subthemes are interpreted in the same way by different researchers.

Double-coding 25% of the interview data is widely considered an acceptable proportion to demonstrate consistency, or to highlight areas of the framework for refinement. The framework categories were continually checked and modified to ensure themes and subthemes were appropriate to index and describe the data fully. A detailed record was kept of the analysis process, including definitions of the themes and concepts and their application. NVivo12™ software was used to manage the data and support analysis.

## Results

### Participants

We conducted 11 interviews. Four participants were BRCA1 and seven BRCA2-positive. Five of the 11 had received or were receiving treatment for breast cancer. Ages ranged from 38 to 77 years. All but one was married or partnered and employed, part or full-time (Table 1).

**Table 1:** Participant characteristics

ID	BRCA status	Time since BRCA diagnosis (approx.) (years)	Cancer diagnosis	Preventive treatment	Age	Relationship status	Employment status
P1	BRCA2	4.5	No	Mastectomy and oophorectomy	54	Married/partner	Part-time
P2	BRCA2	20	No	Oophorectomy	55	Married/partner	Part-time
P3	BRCA1	< 1	Yes	Pending	52	Married/partner	Full-time
P4	BRCA2	4	Yes	None	77	Widowed	Retired
P5	BRCA2	6	Yes	Mastectomy and oophorectomy	46	Married/partner	Full-time
P6	BRCA2	2	No	Mastectomy and oophorectomy	43	Married/partner	Part-time
P7	BRCA1	12	No	Mastectomy and oophorectomy	53	Married/partner	Part-time
P8	BRCA1	5	Yes	Mastectomy and oophorectomy	47	Married/partner	Full-time
P9	BRCA2	7	No	Oophorectomy	44	Married/partner	Full-time
P10	BRCA1	1	No	Oophorectomy	38	Married/partner	Part-time
P11	BRCA2	1	Yes	Oophorectomy	46	Married/partner	Full-time

Two of the interviews were conducted face-to-face and nine by telephone. Over nine hours of interview were recorded and transcribed verbatim. In the following quotes, […] indicates short sections of omitted speech.

### Findings

We identified 18 primary themes and a further 12 subthemes across the interviews. This thematic framework is shown in Table 2. There was considerable interaction and overlap between themes and subthemes and we classified into six overarching themes.

**Table 2:** Thematic framework

Theme	Subtheme
Accessing the system	
Communication breakdown	
Disappointment with follow-up	
Emotional context	
Family communication	
Peer support and learning from the experiences of peers	
Amount and clarity of information provided	
	Related to surgery
	Related to testing and test results
	Relating to psychological support
	Information not provided that participant would have liked
Communication style and delivery of information	
	As experienced
	As preferred or personality dictates
Continuity of care	
Decision-making	
	Certainty relating to surgery
	Certainty relating to testing
	Decision-making and stage of life
	Role of professionals in decision-making
	Uncertainty relating to surgery
	Uncertainty relating to testing
General understanding	
How meetings with HCPs could be improved to aid understanding and experience	
Implications of testing for own and family future	
Influence of family members’ experiences	
Preparedness to receive test results	
Presentation of risk information by HCP	
Temporal and contextual relationship of genetic testing to diagnosis and cancer treatment	
Understanding of and feelings about personal risk	

#### Risk

Risk information was frequently presented as percentages. Personality and individual preferences for receiving information determined how well this approach was received and how participants engaged with risk information. Some participants identified themselves as people who like to have all the information and who process and engage well with percentages in contrast with others who sometimes found this information overwhelming and confusing.

My mum […] just wants to be told what’s the best thing to do and get on with it and she puts her head in the sand. Whereas I need to know my percentage of risk.[P6/43yrs/BRCA2/no cancer]

Not all retained the exact percentages they were told but most were happy to have retained the gist of the discussion rather than the actual numbers presented. Few recalled the use of diagrams or pictures, though the potential benefit of using these to help with understanding was recognised.

They may well have done but, to be perfectly honest, numbers and I don’t mix. It won’t have meant anything other than you’re not at major risk.[P4/77yrs/BRCA2/cancer]

Similarly, some had a rudimentary grasp of the fact that their risk would change over the course of their lifetime but were not able to fully articulate exactly how.

I’ve come away with, at the moment, I’m 65% lifetime risk. They have talked to me about the yearly risk figures, but I get too confused. And I know it’s cumulative, but it doesn’t really mean anything to me.[P10/38yrs/BRCA1/no cancer]

#### Decision-making

Decision-making processes were influenced by the experiences of family as well as evaluation of personal risk based on information provided by HCPs.

I think because I knew that those two people had actually got a gene fault. As soon as I knew I had it, it was like crikey, I just want, I want shot of anything that might put me at the same sort of risk as they had really.[P1/54yrs/BRCA2/no cancer]

Participants did not express uncertainty about the decision to have the genetic test but were sometimes unsure about whether to then have risk-reducing surgery. For some the provision of good screening for breast cancer made the decision more difficult to make as it presented a viable alternative to surgery, whereas surgery was seen as the only feasible option to reduce the risk of ovarian cancer.

With the ovaries it was very clear. This is the best option because we can’t screen you, and that was the message. But with this, I don’t really know whether it will be the right decision or not.[P10/38yrs/BRCA1/no cancer]

For others, the decision around breast risk-reducing surgery was linked to a sense of self and a sense of “being a woman” [P3/ 52yrs/BRCA1/cancer]. A number of participants also noted that their decision might have been different at other stages of life, particularly with reference to having a family.

Five of the participants had received diagnosis and treatment for breast cancer; however, not all were temporally linked with their BRCA diagnosis and subsequent decisions. For those where the processes were contemporaneous, making decisions at the same time could be challenging.

So even though I had the information to hand I was more focused on the breast cancer because that was the reality that we were living with. The other one was if, but maybe.[P3/52yrs/BRCA1/cancer]

A significant subtheme in decision-making related to control and empowerment. Knowledge about BRCA status was empowering and enabled participants to take control of their future in a meaningful way, and risk can be partially mitigated through lifestyle choices enabling a further sense of control.

As my godmother said to me, who also had this gene mutation […] information is power, and if you know you’ve got it you can do something about it [P5/46yrs/BRCA2/cancer]

#### Information and understanding

Participants were generally positive about the amount and clarity of information they received relating to BRCA testing and results, but some were surprised at how much information was presented to them in the first discussion and to be asked to think about the potential of surgery prior to even having their test.

Perhaps you’re better off just having the blood test. And then, saying to you, right, if you test positive for this gene fault, then we invite you to come up here to discuss it all.[P1/54yrs/BRCA2/no cancer]

Most felt prepared to receive their test results, both in terms of having enough information and emotional preparedness. When considering surgical options, participants valued a staged and multi-dimensional approach to information giving. This might include providing DVDs and booklets, a detailed follow-up letter, or having a nurse sit in and/or a dedicated nurse appointment. Some participants highlighted areas of information that they felt had either not been discussed at all or inadequately discussed, including the impact of surgical menopause and the safety of HRT.

I did feel that the communication with the surgeons and the oncologist as well, and always having a nurse in the room is so, so, important. And then, having the letter to follow-up afterwards to the GP, that I get copied on, to explain what it was we talked about.[P8/47yrs/BRCA1/cancer]

#### Communication and improvement

Participants were keen that HCPs adopt an individualised approach to their communication style, ask patients how they prefer to receive information and engage with decision-making, and tailor the discussion in that way. They particularly valued a personal, relaxed, easy to talk to communication style that encouraged them to ask questions.

How do you understand information? How do you make decisions? […] that could be the first thing and then that sets the basis of the relationship.[P6/43yrs/BRCA2/no cancer]

They don’t just tell you they are going to do x, y and z; they involve you. And they listen to what you say.[P9/44yrs/BRCA2/no cancer]

Participants also made practical suggestions to aid the communication process, such as: making it clear in the invitation letter how much would be covered in the initial genetics appointment, and the benefits of bringing someone to that appointment; the benefits of a nurse present at surgical consultations to aid discussion and understanding; and the possibility of flexible ways to continue the discussion after a consultation, such as email follow-up.

I know not everybody has somebody they can go with but that to me, sitting there hearing those risks when you’re just by yourself […] Then having to sit on the train and absorb it all by yourself on the way home.[P1/54yrs/BRCA2/no cancer]

#### Accessing the system: process and frustration

Some participants discussed a struggle to gain access to testing and/or appointments. Once in the system there were occasions where participants felt they had to push for their own follow-up appointments, which can raise anxiety. At least two had appointments cancelled at the last moment or forgotten completely, which was frustrating and upsetting.

I felt like I’ve been frustrated because by the time that I went to the genetic counsellor, I’d been trying for three years to get it.[P6/43yrs/BRCA2/no cancer]

A number expressed disappointment with follow-up, particularly after gynaecological surgery. Some conveyed a feeling of abandonment, with no follow-up and not enough information about effects of surgical menopause and the acceptability of HRT. This was compounded by the perception of insufficient communication between hospital teams and GPs, leaving GPs unsure if they can prescribe HRT.

And now I’ve had my oophorectomy, there’s been no sort of follow-up. Which I suppose there’s no need for it, but I think it would be nice if you could have […] OK, you’ve had this now, you’ve reduced your risk to this, and just a bit more discussion about the next step.[P10/38yrs/BRCA1/no cancer]

Continuity of care and continuity of information provided by the different teams in general was sometimes described as disjointed.

The genetics team believe that the gynaecology team and the breast team speak with each other. And that it's actually like a multi-disciplinary team approach and it's not. And that's a real shame actually because the two in these genetic areas go hand in hand and yet they don't.[P3/52yrs/BRCA1/cancer]

#### Emotional and social drivers

The tentative balance between the weight given to information and discussion provided by HCPS and the influence of family experience and emotional context was evident throughout the interviews.

All BRCA people I think are making decisions in the context of previous experience. We have trauma through multiple diagnoses or deaths or whatever in our families, of other people, which has affected us and we are making our decisions based on that. It’s not just the scientific risk of what our particular gene means to us scientifically and from a biological perspective. It’s what you’ve experienced psychologically also is influencing your decision-making.[P6/ 43yrs/BRCA2/no cancer]

Several issues were raised around family dynamics and communication. While a number of participants saw the benefits of being able to share information and discuss with family members, such an emotive topic can cause friction when one party does not react or respond to the news in the way that the other expects.

You might be interested to talk to my brother […] he’s been avoiding getting tested for about a year now, and I don’t really understand what he’s playing at because he’s got two daughters.[P11/46yrs/BRCA2/cancer]

Not all families are in contact and many are estranged, which can provoke additional feelings of pressure and conflict about how to manage information-sharing. Participants did not suggest that HCPs could or should do anything to facilitate family dynamics, other than perhaps recognise and acknowledge that a standard approach will not fit all families.

So, there was this pressure, I felt, that what if she's got this and she doesn't know and I do, and anything happens to her and I haven't told her then I'm going to be responsible.[P3/52yrs/BRCA1/cancer]

Many participants referenced the importance of peers for emotional support and to ‘fill in gaps’ in the information provided by HCPs and aid decision-making. While more formal, organised support meetings were valued for providing reliable information, participants also discussed the use of Facebook groups for advice.

As far as the geneticist, the genetic counsellor, the Family History Clinic, they’re talking to you on a professional level, which is fine. That gives you the knowledge and the data and the facts, but then you need, sometimes you need a friend in the same boat.[P2/55yrs/BRCA2/no cancer]

## Discussion

We examined the views, experiences and understanding about the genetic testing process and discussions with HCPs of women who were BRCA-positive. We identified 6 overarching concepts: risk, decision-making, information and understanding, communication and improvement, accessing the system: process and frustration, and emotional and social drivers.

Participants valued an individualised approach to communication style which took account of the way they preferred to receive information. This was pertinent particularly when information about risk was presented as percentages, with which not all participants could engage.

For the most part participants were positive about the amount and clarity of information they were given, although the first discussion was sometimes overwhelming, especially for those also managing diagnosis and treatment for breast cancer. For others, the impact of surgical menopause and the safety of HRT were emotive topics which were felt to be inadequately discussed. The need to strike a balance between information content and support for individuals’ processing and comprehension of that information is reported repeatedly in the literature [4–7, 9, 18–20].

Participants used the experiences of, and communication with, family members to evaluate their risk and inform decisions. Numerous studies have reported a strong and complicated relationship between family history and choices made around risk-reducing surgery. For a detailed account, see Padamsee et al. [21]. Peer support, both formal and informal was important emotionally and to ‘fill in gaps’ in the information provided by healthcare professionals and aid with decision-making, consistent with other studies [22, 23].

Women sometimes described the positive test result in terms of ‘empowerment’ and ‘control’, as has been reported elsewhere [24, 25]. This may provide a useful framework for HCPs in their discussions, as a way of enabling women to reframe their uncertainty about risk-management decisions.

### Practical application of findings

The findings from this interview study have been used to inform the TRUSTING training workshops. Verbatim quotes have been used to illustrate and emphasise key points in didactic teaching sessions on risk and uncertainty, and to stimulate associated discussion points. A detailed take-home document was also prepared for workshop attendees, summarising study findings and including direct quotes, to help delegates reflect on their approach to these discussions and their communication style in general.

### Limitations and implications for future research

The study is retrospective in nature; interviewees already had a positive BRCA diagnosis. Time had elapsed since they had been through the process of talking about genetic testing, although discussion and decision-making around risk-reducing surgery was often ongoing. As a result, some did not always remember well the exact processes involved in their referral for genetic testing. Future studies should interview participants in a prospective, preferably longitudinal study.

We are aware of the perspectives that are not represented in these interviews, such as male family members, people who have received a negative test result or findings of a variant of unknown significance or practitioners themselves.

Most of the participants were recruited via BRCA support groups and may represent a different viewpoint to others who do not join a group [26]. One might postulate that people who seek out a support group may have had less positive experiences or be struggling more with decisions than others who do not seek peer support. Reasons for seeking support were not explored with interviewees but would be an interesting comparison in future studies.

We did not set out to identify group differences in the information needs and understanding of women who had and who had not had a diagnosis of breast cancer. However, some women in our study who were managing the process of genetic testing at the same time as their cancer diagnosis and treatment found the process overwhelming, as has been reported elsewhere [27]. Dean et al. [28] suggest that women who are BRCA-positive but have not had a cancer diagnosis have different information needs than those who are currently or have previously been diagnosed and treated for cancer. This may be an important area for future research.

As our primary goal was practical in nature, our sample size was small and our methodological approach did not aim for data saturation; we did not seek to describe all possible experiences of the process of genetic testing and decision-making, rather to highlight recurring, significant themes, valuable to raise in training.

### Conclusion

Using patient narratives in education has been shown to help HCPs better appreciate the patient perspective, enhancing patient-centredness and improving empathy. Incorporating patients’ voices can bring the teaching alive and help HCPs reflect on their approach to these challenging discussions.

These findings have directly informed training materials that will be used and tested in a series of seven workshops with HCPs in the UK. Participants will complete pre and post course assessments to capture any changes in knowledge, confidence and communication skills and provide feedback as to the utility of the training programme.

## Data Availability

The data that support the findings of this study are not publicly available but are available from the corresponding author upon reasonable request.
